# Words for the hearts: a corpus study of metaphors in online depression communities

**DOI:** 10.3389/fpsyg.2023.1227123

**Published:** 2023-08-30

**Authors:** Jiayi Shi, Zhaowei Khoo

**Affiliations:** ^1^School of Foreign Studies, Xi’an Jiaotong University, Xi’an, China; ^2^School of Mathematical and Computer Sciences, Heriot-Watt University, Putrajaya, Malaysia

**Keywords:** metaphorical expressions (ME), conceptual metaphors (CM), MIPVU, semantic domain analysis, online health communities (OHC), depression

## Abstract

**Purpose/significance:**

Humans understand, think, and express themselves through metaphors. The current paper emphasizes the importance of identifying the metaphorical language used in online health communities (OHC) to understand how users frame and make sense of their experiences, which can boost the effectiveness of counseling and interventions for this population.

**Methods/process:**

We used a web crawler to obtain a corpus of an online depression community. We introduced a three-stage procedure for metaphor identification in a Chinese Corpus: (1) combine MIPVU to identify metaphorical expressions (ME) bottom-up and formulate preliminary working hypotheses; (2) collect more ME top-down in the corpus by performing semantic domain analysis on identified ME; and (3) analyze ME and categorize conceptual metaphors using a reference list. In this way, we have gained a greater understanding of how depression sufferers conceptualize their experience metaphorically in an under-represented language in the literature (Chinese) of a new genre (online health community).

**Results/conclusion:**

Main conceptual metaphors for depression are classified into PERSONAL LIFE, INTERPERSONAL RELATIONSHIP, TIME, and CYBERCULTURE metaphors. Identifying depression metaphors in the Chinese corpus pinpoints the sociocultural environment people with depression are experiencing: lack of offline support, social stigmatization, and substitutability of offline support with online support. We confirm a number of depression metaphors found in other languages, providing a theoretical basis for researching, identifying, and treating depression in multilingual settings. Our study also identifies new metaphors with source-target connections based on embodied, sociocultural, and idiosyncratic levels. From these three levels, we analyze metaphor research’s theoretical and practical implications, finding ways to emphasize its inherent cross-disciplinarity meaningfully.

## 1. Introduction

Mental health is a crucial component of public health and a major issue affecting citizens and society. Psychological and behavioral problems have increased due to rapid economic and social development and public health emergencies such as the COVID-19 pandemic. Worldwide, depression is the leading cause of disability, with over 700,000 people dying from depression-related suicides each year, and around 280 million people suffer from depression. In China alone, the number of people with depression has reached 54 million (WHO, 2022). Furthermore, stigmatization and shame associated with depression have resulted in a low identification rate of 21% and a low intervention and treatment rate of 10%, making depression an important public health concern in China (NCMHC, 2022) . Depression might come with symptoms including feeling sad or hopeless, losing interest in activities once enjoyed, having difficulty concentrating, and sometimes thoughts of death or suicide (Leis et al., 2019; Taylor-Jackson and Moustafa, 2021). These symptoms can make it difficult to leave the house, attend outdoor activities, or even socialize with others. Self-isolation and disconnection can lead people with depression to ruminate more on negative thoughts and feelings, thus worsening their symptoms (Yoon et al., 2019; WHO, 2022).

Online health communities (OHC) have changed how people access health information. Users in OHC talk about their conditions and treatment options, share their cases and experiences, seek social support and help, and alleviate social fears and stigma (Xu and Zhang, 2016; Ríssola et al., 2019). A better understanding of how OHC users with depression communicate can boost the effectiveness of counseling and interventions for this population by shedding light on how they frame and make sense of their experiences. However, this line of research is still in its infancy, and identifying and analyzing more ambiguous and abstract information, such as metaphors, remains challenging (Kauschke et al., 2018; Poppi and Kravanja, 2019; Coll-Florit et al., 2021).

The current paper emphasizes the importance of identifying the metaphorical language used in OHC to gain insights into their cognitive mechanisms and behavioral patterns. Such insights could help identify and manage mental health issues, particularly in cultural contexts where individuals are reluctant to seek help due to the stigma associated with depression. First, we will review metaphor identification research and describe its gradual focus on combining metaphors in language, thought, and communication. Afterward, we present an overview of metaphor analysis in health-related communication, emphasizing the need to examine metaphor use in OHC, in our case, among depressed users. In the next stage of the paper, we identify and analyze depression metaphors in a Chinese corpus by following a three-stage procedure. This paper concludes with methodological, theoretical, and practical implications and suggestions for future research, synthesizing psychotherapy and discourse analytic perspectives.

## 2. Literature review

### 2.1. Metaphor identification research

By extending metaphors beyond rhetoric to cognition, Conceptual Metaphor Theory (CMT) marks the cognitive turn in metaphor study (Lakoff and Johnson, 1980; Lakoff, 1993). By focusing on using metaphors as a cognitive tool for understanding and organizing experience (Lakoff and Johnson, 1980), CMT departed from the traditional belief that metaphors are ornamental, arguing metaphors are of conceiving one thing in terms of another (Lakoff and Johnson, 1980). A cognitive linguist describes conceptual metaphors (CM) according to the formula TARGET IS SOURCE, i.e., CM allow one to conceptualize a target domain through a source domain by comparing properties and categorical relationships. This process of comparing and categorizing metaphors is also known as metaphor processing (Tong et al., 2021). Linguistic metaphors stem from CM or cross-domain mappings within one’s conceptual system. For example, DEPRESSION IS BATTLE maps the abstract concept of depression onto the concept of battle. This metaphor allows us to understand depression in terms of conflict, violence, and victory. Metaphorical expressions (ME) are prevalent in everyday life as evidence that linguistic expressions (metaphors within language) reflect certain cognitive systems (metaphors in thought) (Charteris-Black, 2012; Coll-Florit and Climent, 2019).

Researchers have questioned the legitimacy of extrapolating linguistic expressions to cognitive structures and have argued that language users may not similarly relate to these ME in their minds (Semino et al., 2004, 2016), i.e., metaphorical languages do not necessarily produce metaphorical cognition, and the same metaphor can also produce different cognitive patterns or metaphorical systems in different groups (McMullen and Conway, 2002; Reijnierse et al., 2017). Metaphor processing occurs during text comprehension, leading to the emergence of features that are not inherent in target or source domains, also known as emergent meaning (Tong et al., 2021). For example, *depression is battle* contains far richer shades of meaning than *depression is hard*. Both express a similar meaning, but the latter expression is unlikely to be as concise as the use of the word *battle*. *Depression is a battle* denotes depression is a struggle, a fight, and a test of endurance. These additional connotations add richness and depth to the understanding of depression. Language users might deliberately introduce topic shifts or perspective changes into discourses (Reijnierse et al., 2017). For instance, looking at depression from the perspective of a battle can help us see it differently, not as something to be ashamed of, but rather as a challenge to overcome. It is necessary then to include an analysis of metaphors in the communicative process (metaphors in communication) (Reijnierse et al., 2017; Coll-Florit and Climent, 2019).

As a result, metaphor research has developed a three-dimensional analytical framework: metaphors in language, thought, and communication (Semino et al., 2016; Coll-Florit and Climent, 2019; Tong et al., 2021). A direct result of this development of metaphor theory is the development of metaphor identification methods as well.

Initially, metaphor studies sought to establish a systematic correspondence between metaphors in language and thought. Metaphor identification aimed to analyze the conflict between metaphorical and literal meanings in non-textualized example sentences, leaving metaphor identification largely to subjective judgments (Gibbs and Franks, 2002; Semino et al., 2004; Kimmel, 2010). In the same way as (1), if the word *freedom* evokes a concept directly related to slavery and imprisonment, then its use may be considered metaphorical, whereas a more general interpretation of the concept of *freedom* that entails the ability to choose points to a literal meaning (Steen et al., 2010).

(1) They had so kindly offered *freedom* (Coll-Florit and Climent, 2019).

The MIP (metaphor identification procedure) proposed by the Pragglejaz Group (2007) provides normative steps for metaphor identification and greatly simplifies the problem by emphasizing attention to the distinction between contextual meaning and basic meaning. The basic meaning is an important concept in metaphor identification systems. It tends to be more concrete, historical, and more likely to evoke human sensory experience. Suppose a lexical unit has a more basic current meaning in other contexts than in the given context, and the contextual meaning contrasts with the basic meaning. In that case, the lexical unit is labeled as a metaphor. The basic meaning derives from the dictionary definition (Burgers et al., 2016). In (2), for example, *attack* denotes verbal exchange in the current context; however, this is a non-direct meaning because the basic meaning of *attack* in other contexts is physical activity; and through this layer of basic meaning, the current context implies the meaning of verbal exchange. Thus the *attack* in example (2) is a ME.

(2) Hillary Clinton *attacks* Bernie Sanders as New York primary looms (Semino et al., 2004).

Informed by CMT, Steen et al. (2010) propose the Metaphor Identification Procedure Vrije Universiteit (MIPVU) for manually identifying metaphor-related words (MRWs) in a text. MIPVU’s comparison of basic and metaphorical meanings makes metaphor identification less dependent on researchers’ subjective judgment and allows for more objective identification of the role of metaphors in communication. There are also other important works for metaphor identification and interpretation. The Deliberate Metaphor Identification Procedure (DMIP) provides a useful framework for understanding how people deliberately use metaphors to introduce topic shifts or perspective changes into discourses (Reijnierse et al., 2017). DMIP presents normative procedures for metaphor identification and strengthens the function of metaphors as their purpose in communication (e.g., to persuade, to add interest). For example, DEPRESSION IS BATTLE provides a frame of reference for understanding depression in terms of conflict and victory, and its purpose is to persuade the reader that depression is a serious illness and hard to cope with. Researchers also focus on systematicity, which refers to the idea that metaphors are not just isolated figures of speech, but systematically based on underlying conceptual structures through recurrent connections between the source and the target (Sardinha, 2011; Van Stee, 2018). For example, DEPRESSION IS BATTLE is based on the underlying conceptual structure of conflict, including elements such as opponents, strategies, and outcomes. Similar metaphors, such as depression is WAR, CONFLICT, and FIGHT, are in the same part of a larger system of metaphors that conceptualizes depression in terms of conflict.

These works provide valuable insights on metaphor identification and interpretation methods, especially the lexical patterning of metaphorical language and criteria for determining metaphor use. However, they require too much manual labor and are time-consuming (Dorst et al., 2013; Marhula and Rosinski, 2017; Landau et al., 2018). Moreover, Gibbs (2008) points out that claims regarding the importance or ubiquity of particular metaphorical patterns lack empirical basis. For example, one could report the frequency of different metaphors appearing in specific texts or compare metaphor analysis with that from large corpora (p. 12).

In this respect, corpus analysis, which describes linguistic properties through large-scale data, provides explanatory power to metaphor theory, since linguistic expressions (metaphors in language) corroborate cognitive systems (metaphors in thought). As a result of using corpus analysis to quantify metaphorical patterns, CM identified by introspection in cognitive linguistic analyses are substantiated, thereby contributing to our understanding of CM’ relative salience in communication (metaphors in communication) (Semino et al., 2016; Coll-Florit and Climent, 2019; Tong et al., 2021).

There are mainly two approaches to corpus analysis of metaphors. Using a bottom-up approach requires the researcher to identify all metaphors in a text and analyze them, but the vast amount of information makes it impossible. A top-down approach begins with a list of known metaphors, then the researcher searches for those metaphors in the text and adds a metaphor that is not already there. However, identifying unusual or creative metaphors is often difficult since it only identifies metaphors already known to the researcher. Researchers have minimized the risk of overlooking potentially interesting patterns and avoided the disadvantages of both approaches. Sardinha (2011) suggests adopting a preselection procedure to limit the number of concordances. In Semino (2017), metaphors are studied qualitatively, including identification, interpretation, and explanation, and afterward, concordances are quantitatively analyzed. Charteris-Black (2012) analyses ME using keywords that previous research had shown are commonly used metaphorically by people experiencing depression, e.g., DARKNESS, DESCENT, and WEIGHT. As a next step, he determines whether these keywords had a more basic meaning using the Pragglejaz Group (2007) method in the concordance context. Coll-Florit et al. (2021) incorporate both approaches and uses working hypotheses and reference lists to identify metaphor patterns.

There is a strong focus on English instead of other languages in corpus analysis of metaphors so far. Unlike English, Chinese uses a morphosyllabic writing system in which the basic orthographic unit is the character. There is a phonological syllable in each character, which corresponds in most cases to a morpheme. Chinese words can contain multiple characters; without explicit word separators, any two or more consecutive characters can represent a single word or phrase. Chinese is also known for having relatively simple word forms, with compound words being the most common (Pritzker, 2003; McEnery and Xiao, 2004; Tay, 2015). As a result, certain word classes are also difficult to distinguish from one another (e.g., verbs and adjectives) in the absence of morphological clues. Considering the Chinese orthographic and morphological differences above, delineating units of analysis may be challenging.

Our study aims to address the problem of metaphor identification in a large-scale Chinese corpus. We seek to develop a more reliable, efficient, and replicable metaphor identification procedure for Chinese corpora, combining bottom-up and top-down approaches.

### 2.2. Metaphors of depression

Metaphors are important for human beings to recognize, think and express. Comparing uncertain and unfamiliar experiences to familiar references helps people communicate complicated, sensitive, and emotional topics (Gibbs, 2008). A metaphor is a form of figurative language that understands a target domain (which often refers to a complex or emotional experience) by a source domain (which tends to be an object or phenomenon that is simpler and more familiar than the target domain). For example, people who suffer from depression might describe their TREATMENT TRAJECTORY (i.e., the target domain) as JOURNEY (i.e., the source domain) to convey the varied emotions they experienced at different points in the process. Healthcare professionals benefit from using metaphors in counseling and psychotherapy in challenging situations (Tay and Neimeyer, 2021). In addition to helping people understand their symptoms and treatment options, metaphors can help people cope with the emotional challenges of chronic illness. Some recent studies show metaphor use in various medical conditions, such as cancer (Semino et al., 2015; Spina et al., 2018; Hendricks et al., 2019), diabetes (Abdollahi et al., 2019), end-of-life services (Potts and Semino, 2017), and COVID-19 (Panzeri et al., 2021; Semino, 2021; Pérez-Sobrino et al., 2022).

In the mental health arena, researchers from both psychotherapy and linguistics share their insights on metaphors as a tool to facilitate clients’ communication with therapists (Tay, 2017). Psychotherapists study metaphors from a functional perspective and need epistemological and communicative competencies to achieve client compliance. Using metaphors can make client compliance more feasible by explaining the reason for diagnosis and treatment and framing the experience of illness according to the client’s needs. Alternatively, metaphors may encourage clients’ beliefs about illness and allow them to make a shared decision with their therapists. Through metaphors, clients can better understand their emotions, thoughts, and behaviors (Pritzker, 2003; Tay, 2015; Tay and Neimeyer, 2021).

Meanwhile, linguistics researchers focus more on metaphors’ identification and contextual richness (McMullen and Conway, 2002; Charteris-Black, 2012; Poppi and Kravanja, 2019). In particular, they are interested in metaphors’ target and source domains. For instance, Barcelona (1986) conducted one of the earliest qualitative studies on depression metaphors based on his experiences (Coll-Florit et al., 2021). He exhibited some metaphors that would later be documented in empirical studies to structure depression in English, including the directional metaphor HAPPINESS IS UP/UNHAPPINESS IS DOWN (e.g., “I am in low spirit”), and the perceptual metaphor HAPPINESS IS LIGHT/UNHAPPINESS IS DARKNESS (e.g., “I feel under a cloud”). Depression has been conceptualized as FORCE, BURDEN, JOURNEY, LIVING ORGANISM, ENEMY, or BOUNDED SPACE.

Researchers only began analyzing metaphors of depression through corpus analysis of larger corpora in recent decades because it is difficult to collect a large corpus of text from people with depression. McMullen and Conway (2002) analyze the therapy session recordings of 21 people diagnosed with major depression and find that the dominant conventional metaphor for depression is DESCENT. It is often described as an expression of physical descent, and the destination is envisioned as a container (such as a well). According to Semino (2017), the most frequent domains for clinical depression are UP/DOWN, ENCLOSED SPACE, JOURNEY, and PHYSICAL ENTITY. Depression sometimes equates with ENEMY and depression sufferers are conceptualized as MALFUNCTIONING MACHINES. Charteris-Black (2012) analyze 38 interview discourses to determine whether depression is gendered, and they find the dominant conventional metaphor of depression is DESCENT. Men and women use similar metaphors (DESCENT, WEIGHT, PRESSURE, DARKNESS, and LIGHT). Within their corpus, containment metaphors rank second in frequency. The result is a model for depression in which the self is “contained” within the depression, but also “contains” the trapped, sad feelings.

Furthermore, most current research pertains to English culture, when other cultures are also worthy of scholarly interest. An individual’s culture profoundly affects how they perceive and express their experiences, thoughts, and feelings (Lakoff, 1993; Gibbs and Franks, 2002; Kimmel, 2010; Semino et al., 2015; Munday et al., 2021). As Lakoff and Johnson (1980) noted, “the most fundamental values in a culture will be coherent with the metaphorical structure of the most fundamental concepts in the culture” (p. 22). Using interviews with three depressed people, Yu (1995) demonstrates the Chinese metaphor of thinking and feeling is characterized by the heart, because the Chinese believe that the heart and the brain are intimately connected, regardless of the primacy of the heart, and that any damage to the heart can negatively affect the brain on a cognitive and emotional level. Coll-Florit et al. (2021) examine 23 blogs in Catalan by people with depression. They suggest that contextual factors (such as stigma, poor communication, or medical practices perceived as repressive) can also significantly impact the lives of people with depression.

To fill these gaps, the current study addresses a need for research on metaphors about depression which can inform practice and contribute to our understanding of how people experience, make sense of, and communicate about depression in a context where people are still reluctant to discuss their depression openly, for fear of being judged or ostracized. By understanding how online health community users frame and make sense of their depression through metaphors, we can boost the effectiveness of counseling and interventions for this population.

To pursue these aims, we posed the following research question:

*RQ*: For making sense of and communicating about their experiences with depression, what metaphors do OHC users use?

## 3. Research methodology

**Figure 1:**
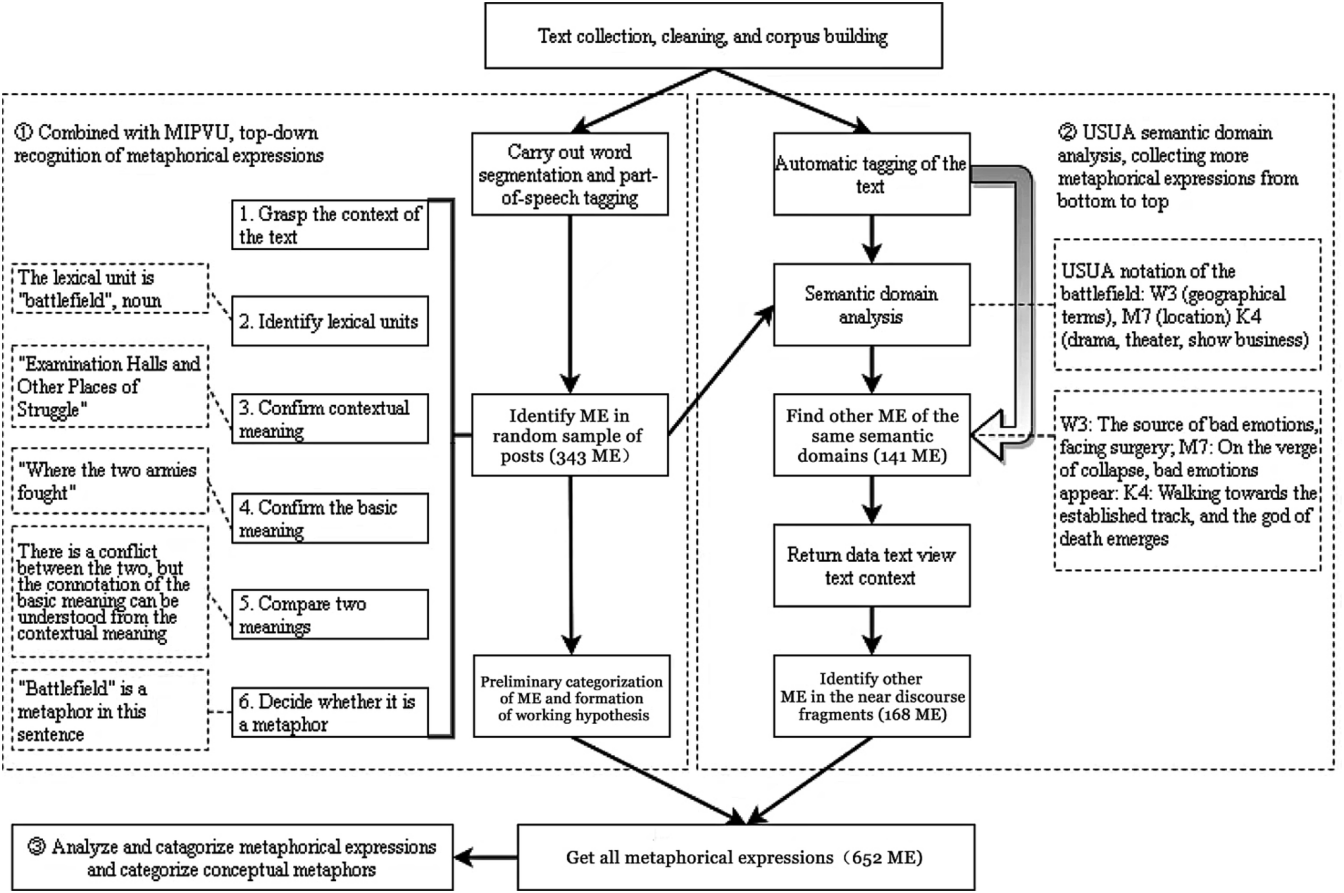
Stages of metaphor identification procedure in the Chinese corpus. Uses the example (3) to explain the key stages of the procedure: (3) 只要/vu 闭上/v 双眼/n 便/a 不会/vu 再/d 失去/v 任何/r 事物/n ，/w 即便/c 如此/r ，/w 你/r 还是/d 要/vu 投身/v战场/n 吗/u? (As long as you close eyes will not lose anything again, even if so, you still want to join the battlefield?).

The following section explains our research methods in details. Using a web crawler, we extracted texts from an online community of depression sufferers. A three-stage procedure is followed to identify metaphors within the self-compiled corpus, including a manual bottom-up metaphor identification process, an automatic, top-down semantic domain analysis, and a reference list for categorizing metaphors (see Figure 1).

(1) Bottom-up identification of ME and the initial formation of working hypotheses in conjunction with MIPVU: 343 metaphorical expressions (ME) are manually identified, resulting in preliminary working hypotheses of CM of depression.
(2) Collect more ME in the corpus from top-down by performing semantic domain analysis on the identified ME: Using USUA semantic domain analysis on the 343 ME identified in stage 1, 141 ME within the same semantic domains are identified, and another 168 extended ME are identified in the near discourse fragments.
(3) Use the reference list to analyze ME and categorize CM.

Using comparison and categorization as paths, this procedure explores a variety of target-source pairs: the bottom-up analysis helps in finding metaphors based on their comparable similarities, whereas the top-down semantic domain analysis helps in tracing metaphors from their categorization paths.

### 3.1. Data collection and analysis

Since its launch in August 2009, Sina Weibo has been experiencing explosive growth, with 511 million monthly active users and 224 million daily active users as of early 2021, making it one of China’s largest social networking platforms (SINA, 2021). Users access it through multiple mobile terminals such as personal computers, cell phones, and tablets to instantly share and spread interactive information through text, pictures, videos, and other multimodal data.

In this study, we used Python to obtain the open-source data on Weibo. Using “depression” as the keyword, 3,854 posts were collected from January 1, 2022, to March 31, 2022, on Sina Weibo’s Depression Super Talk platform. The timeline data of each user included: post text, username, posting time, retweeting times, comments, etc. After eliminating short texts that contain too little useful information (<7 words), we kept 2,664 posts. After data cleaning of the texts, a corpus of 23,195 characters was built.

### 3.2. Chinese corpus metaphor identification procedure

#### 3.2.1. Stage 1: bottom-up identification of metaphorical expressions using MIPVU

The Pragglejaz Group (2007) emphasizes the need to use a dictionary compiled based on large-scale corpus analysis as a reference to distinguish between basic and contextual meanings. Accordingly, we selected the Modern Chinese Dictionary (7th edition) and the Xinhua Dictionary (12th edition) (TCP, 2020) as the lexical tools of the project. Both are the most authoritative Chinese dictionaries available, continuously revised and reprinted according to language norms and standards, with the former containing nearly 70,000 entries and the latter 13,000 single words. The advantage of the former is that the examples of words are longer and rich in contextual information, which facilitates the determination of contextual meaning. The latter’s advantage is its introduction of the words’ historical meanings, which can be used as basic meanings in MIPVU. In addition, we used the Modern Chinese Corpus of the National Language Commission (Xiao, 2016) as a reference, being it a large-scale balanced corpus with a wide range of categories and PoS (part of speech) coding of the corpus.

Using MIPVU for Chinese language identification requires lexical unit adjustment. While English has strict word boundaries, Chinese has blurred word boundaries as described in section 2.1. Chinese is also known for its relatively simple word forms, and the distinction between word classes is also very blurred. These have motivated computational linguists to adopt different standard word separation algorithms (Pritzker, 2003; McEnery and Xiao, 2004; Tay, 2015). Using the BFSU Standard Parser developed at Beijing Foreign Studies University, two native Mandarin-speaking coders reviewed the output and corrected a few tagging errors (e.g., proper nouns, idioms). Two coders randomly selected 300 posts and compared the basic and contextual meanings for manual metaphor identification based on the definitions given by two dictionaries.

We explain step-by-step the process of MIPVU (6 steps in total) for metaphor identification using (3) as an example:

Step 1: Read through the entire text to fully grasp the context and meaning of the text.

Step 2: Establish the lexical unit for the analysis on the result of the text tagging. The BFSU Standard Parser is used to identify lexical units, especially adjectives, adverbs, nouns and verb word classes (part of speech, PoS). The lexical unit established here is战场 *battlefield*, a noun labeled as /n (noun) by the BFSU Standard Parser.

Step 3: Refer to the context to establish the contextual meaning of the lexical unit. For each lexical unit in the text, check its meaning in the context, i.e., how it applies to the entity, relationship, or attribute in the current situation evoked by the text (contextual meaning). Consider the content before and after the lexical unit. The contextual meaning is derived from dictionary interpretations. After comparing the interpretations given in the dictionaries, the more refined interpretation of the latter was selected: 2. 考场及其他斗争的场所*the examination room and other places of struggle*.

Step 4: Analyze the presence of more basic interpretations. Contrary to the contextual meaning, which can be abstract, the basic meanings are more concrete, related to bodily actions, more precise (as opposed to vague), and historically older. Based on the dictionary, the basic meaning of *battlefield* is: 两军交战的地方*A place where two armies are engaged in battle*.

Step 5: Decide whether the contextual meaning conflicts with the basic meaning, but can be understood by comparison. The basic meaning of *battlefield* is more concrete and historical than the contextual meaning that indicates the place of struggle, and the two meanings are in conflict. Nevertheless, it is still possible to understand the situation and emotion of struggle from the contextual meaning.

Step 6: Therefore, code 战场*battlefield* as a ME.

Two coders analyzed 300 randomly selected posts and annotated them with NVIVO software, yielding 343 ME with rater reliability of Cohen’s Kappa of 0.71. (2007) uses the Kappa values reported by previous MIP and MIPVU raters’ reliability as an annotation criterion for metaphor identification, reporting a Kappa value of 0.62 for dialogue texts and 0.72 for news texts, while Steen et al. (2010) report Kappa values ranging from 0.70 to 0.96 for different text types (academic text, fiction, news, and dialogue), with the dialogue type obtaining the lowest value. Therefore, Chinese metaphor identification using MIPVU meet the rater reliability criteria.

We performed NVIVO coding of 343 ME to classify metaphors into preliminary target domain categories, such as PEOPLE WITH DEPRESSION, DISORDER, LIFE WITH DEPRESSION, and INTERPERSONAL RELATIONSHIP. This division facilitates the formation of our initial working hypothesis (working hypothesis) (De Choudhury et al., 2013; Tobin and Lyddy, 2014; Yoon et al., 2019). Since we analyzed a smaller amount of extracted data (300 randomly selected posts), instead of analyzing each word using the MIPVU, the time required is significantly reduced. We might miss some ME candidates. However, the study aims not to detect every ME in the corpus, but to identify the main CM; as elaborated in section 2.1, to achieve an awareness of the systematic linguistic correspondence behind ME provides explanatory power to metaphor theory. The substantial time savings offset the deficit of losing some ME candidates to ensure the project’s feasibility.

#### 3.2.2. Stage 2: top-down ME identification with semantic domain analysis

The second phase of the study used USUA Semantic Tagset, an inbuilt tagset of an online corpus tool (Wmatrix) developed by Lancaster University, to automatically annotate the semantic domains in the corpus (Rayson, 2008; Piao et al., 2015). 21 major discursive fields or semantic domains (A-Z) have been assigned to words. Semantic tags group together similar word senses because they are connected at some level with similar mental concepts (for a complete list and prototypical examples).^1^ Each semantic domain contains several sub-domains, 232 in total: e.g., the semantic domain “S social behavior, state, and process” contains “S1 social behavior, state; S2 people; S3 relationships; S4 relatives …S8 help/hindrance; S9 religion, superstition,” etc., and the sub-domains can divide further, e.g., “S3 relationships” has sub-domains “S3.1 General relationship, S3.2 Intimacy/sexual relationship.” This list includes synonyms, antonyms, hypernyms, and hyponyms, with approximately 37,000 words in the lexicon and over 16,000 multi-word units in the template list.

The specific analysis process is to perform semantic domain analysis on the ME identified in Stage 1 (see section 3.1), find other lexical units under the same semantic domain, and check the concordance context (*cf.* Semino, 2017). We analyzed only lexical units of depression-related expressions, which saved a lot of time since many lexical units are under one semantic domain. From a top-down approach, searching lexical units through semantic domains facilitates metaphor identification. Though the process is automated, the user can review alternative tags for words with ambiguous meanings. Based on its built-in dictionaries, the USUA tagging system provides alternative tags in descending probability order. As a simple example, the system assigns the construct ‘DEPRESSION IS A BATTLEFIELD’ to the category ‘W3 geographic terms’, along with ‘M7 location’ and ‘K4 drama, theatre, performing arts’. The USUA tagging system has identified ‘battlefield’ as a noun in each case, providing the word’s most common meaning: space, place, and fight.

We repeated the MIPVU identification procedure to determine whether the lexical unit is a ME in the discourse fragment. In return, we found more ME used for depression in the categories of W3, M7, and K4, such as:

W3 Geographic terms:

(4) 所有的坏/情绪的源头我妈能占一半。
*All of bad emotions **source** my mom can take up half.*

(5) 希望这辈子不用再面对手术。
*Hope this lifetime no need again **face** surgery.*

(6) 我情绪崩溃想消失。
*My emotions collapse and (I) want to **disappear.***

M7 Location:

(7) 一些细节总是能把自己逼到崩溃的边缘。
*Some details can always force me to the **edge** of collapse.*

(8) 总会有莫名其妙的情绪出现把我打倒。
*Always have some emotions **appear** that beat me down.*

(9) 你能看见我吗, 我把我弄丢了。
*Can you **see** me, I **lost** myself.*

K4 Drama, Theatre, Performing Arts:

(10) 原生家庭深深地影响了我, 我出生在这泥沼里，挣扎了很久也没**逃**出去。
*My family of origin deeply affected me, I was born in this mire, struggled for a long time but did not **escape.***

(11) 本来可以安安稳稳朝着既定**轨道**向前走，为什么会这样?
*Could have peacefully walked toward a steady **track**, why would be so*?

(12) 我的未来又**浮现**出了死神的怀抱。
*My future again **emerges** The Reaper’s embrace.*

Using the USUA semantic domains for top-down metaphor identification, we found 141 more ME with the same semantic domains of the 343 ME identified in stage 1. Discourse-level information helps to identify extended metaphors, i.e., sustained use of the same metaphors in a discourse fragment. As a result, we further identified 168 extended metaphors in near discourse fragments (after removing duplicate items), such as (4) 占 *take up*, (6) 奔溃*collapse*, (7) 逼到*force to*, (8) 打倒 *beat down*, (10) 沼泽*mire*, 挣扎*struggle*, (11) 向前*forward*, (11) 走*walk*, (12) 死神*The Reaper* and 怀抱 *embrace*. In total, we identified 309 ME in the second phase.

Semantic domains are very useful in formulating patterns, which have been introduced in corpus research of metaphors based on the similarity between the source and the target (Tay, 2017; Spina et al., 2018). Similarity builds on the different levels of “sameness” between the source and the target, which makes it possible to link two things psychologically (Semino, 2017). It essentially involves reorganizing categorical relations. Metaphors are created by reorganizations within categories (*cf.* section 2.1). Semino et al. (2016) suggest that the USUA semantic domains effectively identify metaphors through categorical similarities. By specifying the semantic grouping rather than the individual words, they can be more useful when used as search terms to query a corpus. A disambiguation phase consists of seven dimensions: the POS-tag, the general likelihood ranking, the overlapping template resolution, the domain of discourse, text-based disambiguation, contextual rules, and local probabilistic disambiguation (Rayson, 2008). To determine the most likely semantic tag, the USAS-tagger uses information such as the grammatical tag, frequency of semantic sense in the reference data, the known domain of the surrounding discourse, and the assumption that a word carries a consistent semantic meaning throughout a text. Regarding accuracy, the Wmatrix USAS has high reported figures (96–97 and 91%, respectively) (Collins, 2011; Piao et al., 2015; Semino et al., 2015).

Using Wmatrix across research domains demonstrates its usefulness as a ready-made analytical tool, but we must be cautious about accepting USAS categorization as standard. Analyzing how such a system privileges certain readings of the text is worthwhile. In most cases, researchers use semantic tagging as a preliminary step in their analysis before exploring the findings in greater depth (Collins, 2011). In example (6) *disappear* and (8) *appear*, despite their opposite meanings, they are classified as Geographic terms and Locations, respectively. As a further consideration, we classified (6) *disappear* in People With Depression as CONTAINER because it contextualizes the speaker’s feeling of isolation and constrained feelings, which is characterized by being constricted by bad emotions and feeling isolated. Our categorization of (8) *appear* is DEPRESSION IS LIVING ORGANISM because bad emotions are personified as beings that show up and beat down the patient. In another example (5), USUA automatically classifies *face* as metaphors in Geographic terms. The patient is reluctant to live with surgery, which makes TREATMENT OPTION IS LIVING ORGANISM a more accurate classification of the ME.

#### 3.2.3. Stage 3: metaphor reference list: analyze ME and categorize conceptual metaphors

We used a reference list to analyze ME and categorize conceptual metaphors. Specifically, the list contains the following studies:

(1) Literature on depression metaphors (Hughes-Hammer et al., 1998; McMullen and Conway, 2002; Pritzker, 2003, 2007; Schoeneman et al., 2004; Charteris-Black, 2012; Refaie, 2014; Kauschke et al., 2018; Leis et al., 2019; Yoon et al., 2019; Beck, 2020; Tong, 2020; Coll-Florit et al., 2021; Malkomsen et al., 2021; Munday et al., 2021; Taylor-Jackson and Moustafa, 2021)
(2) List of metaphors (Lakoff et al., 1991)
(3) Oxford guide to metaphors in CBT (Stott et al., 2010)

In this study, the reference list is used in two ways. First, the reference list allows us to locate CM quickly, combining metaphors in language, thought, and communication. The reference list contains works that provide valuable insight into metaphor identification and interpretation techniques, especially those that discuss the lexical patterning of metaphorical language and how to determine whether a metaphor is being used. Some typical depression metaphors have been illustrated in the literature, including DEPRESSION IS CONTAINER, FORCE, BURDEN, JOURNEY, and LIVING ORGANISM. We first look at the metaphor reference list to see if a corresponding CM exists. We then verify or counter-verify them, resulting in more consistent and time-saving annotations. The main reason is that many generic CM in the reference list are repeated in texts. For instance, DISORDER IS CONTAINER can be instantiated in a mire, well, hell, whirlpool, jar, and swamp. The reference list also contains a guideline for metaphors in cognitive behavioral therapy to help us understand metaphors as a means of communication (metaphors in communication). As the guideline describes (Stott et al., 2010), isolation and withdrawal are core beliefs and schemas of depression (p. 112–114). Cognitive and behavioral factors conspire to maintain isolation: People suffering from depression often feel isolated, develop a habitual pattern of low activity, and become truly isolated. This helps us understand DEPRESSION IS CONTAINER and DEPRESSION IS THEATRE, as the metaphorical essence of both is depressed individuals being cut off by a barrier and losing human connection with others.

The second benefit is that the reference list provides a consistent and standardized way to categorize CM, allowing for a better understanding of the topic and provides a framework for analysis. In example (19), we categorize *rope* as a THING, but it could be interpreted as an animated entity or personification based on the context (“binds me tightly”). Regarding the current research, the distinction is particularly relevant and we categorized based on literature preferences derived from the compiled reference list. For instance, Coll-Florit et al. (2021) differentiate DISORDER IS THING and DISORDER IS LIVING ORGANISM as the former “usually is a nuisance” (p. 11), while the latter “is instantiated in terms of a monster, an evil being or ghost, or a person with whom you live, or to whom you attribute characteristic human actions” (p. 10). Based on online visual representations, Poppi and Kravanja (2019) distinguish between DEPRESSION IS THING (e.g., tentacular entities) features in the victim’s inability to change and immobility, while DEPRESSION IS LIVING ORGANISM (e.g., ferocious animals) portrays predatory or hostile traits of depression. Therefore, we categorized (19) *rope* as DEPRESSION IS THING since it denotes discomfort and nuisance. In contrast, we categorized (5) *face* as TREATMENT OPTION IS LIVING ORGANISM, for the patient expresses reluctance to live with surgeries as if they were people.

## 4. Results

We identified a total of 652 ME. Drawing on the principle of grounded theory, we used NVIVO to classify them into four types: PERONSAL LIFE, INTERPERSONAL RELATIONSHIP, TIME, and CYBERCULTURE. Table 1 shows the types and their numbers of ME. 77.15% of the ME belong to PERONSAL LIFE, which involves how users understand their lives, themselves, the disorder, and treatment options. INTERPERSONAL RELATIONSHIP metaphors accounted for 12.27% of the total. In addition, TIME and CYBERCULTURE metaphors accounted for 6.29 and 3.07%, respectively.

**Table 1:** Categories and number of metaphorical expressions of depression.

Metaphorical categories	Quantity	Percentage
PERSONAL LIFE *metaphors*	503	77.15%
DISORDER is	230	35.28%
---DESCENT	65	9.96%
---DARKNESS	41	6.29%
---CONTAINER	32	4.91%
---WEIGHT	28	4.29%
---COLDNESS	21	3.22%
---LIVING ORGANISM	20	3.07%
---FORCE	11	1.69%
---THING	9	1.38%
---IMBALANCE	3	0.46%
LIFE WITH DEPRESSION is	143	21.93%
---JOURNEY	58	8.90%
---WAR	44	6.75%
---THEATRE	32	4.91%
---CONTAINER	7	1.07%
---THING	2	0.31%
PEOPLE WITH DEPRESSION are	102	15.64%
---CONTAINER	34	5.21%
---MACHINE	28	4.29%
---THING	25	3.83%
---SPLIT SELF	8	1.23%
--- THEATRICAL ROLE	7	1.07%
TREATMENT OPTION	28	4.29%
---SUPPORT	16	2.45%
---FORCE	5	0.77%
---LIVING ORGANISM	4	0.61%
---CAPTOR	3	0.46%
INTERPERSONAL RELATIONSHIP *metaphors*	80	12.27%
---REPRESSIVE POWER	32	4.91%
---ENEMY	24	3.68%
---CONTAINER	14	2.15%
---SUPPORT	10	1.53%
TIME *metaphors*	41	6.29%
CYBERCULTURE *Metaphors*	20	3.07%
Other	8	1.23%
	652	

### 4.1. PERSONAL LIFE metaphors

The four main target domains of personal life metaphors are DISORDER, LIFE WITH DEPRESSION, PEOPLE WITH DEPRESSION and TREATMENT OPTION.

DISORDER metaphors are the most frequent (35.28%), related to pleasure deficit and physical ailments. Most DISORDER metaphors are embodied metaphors (30.82%), including (13) DESCENT, (14) DARKNESS, (15) WEIGHT, and (16) COLDNESS. According to CMT, conceptual metaphors help us understand abstract concepts by using the sensory-motor system as a representation. It is almost universal to metaphorically understand abstract targets in terms of everyday bodily experiences, such as warmth, hunger, walking toward a destination, and moving in and out of physical containers (Wen and Hua, 2018). In the Chinese corpus, although DEPRESSION IS DESCENT does not appear as frequently as it does in McMullen and Conway (2002) or Charteris-Black (2012), metaphors related to the loss of control over emotions are quite common. As shown in Table 1, DESCENT (9.97%) is the main CM to describe the disorder. This result is consistent with the embodied conceptual association of HAPPINESS IS UP/UNHAPPINESS IS DOWN. For instance, as noted above (*cf.* section 2.2), in the English context, McMullen and Conway (2002) analyze the therapy session recordings of 21 people diagnosed with major depression and find that 90% of conventional metaphor for depression is DESCENT. In our corpus, DESCENT metaphors relate closely to (13, 17) CONTAINER metaphors (4.91%), in which individuals experience a physical descent, and the destination is envisioned as a container (such as 漩涡 *whirlpool*, 沼泽 *mire*, 深井*deep well*, 水坑*pit*, 地狱 *hell*).

(13) 我就只能自己苦笑着自嘲着在这个情绪**漩涡**里一点一点**沉底**
*I can only bitterly smile and mock myself as I slowly **sink** to the **bottom** in this emotional **whirlpool**.*

(14) 大脑里的灯全部被人关掉，一片**黑暗**，就像被关在没有窗户密闭的空间就像溺水的人掉进水坑。
*All the lights in my brain have been turned off by someone, it’s **dark**, like being locked in a windowless enclosed space, like a drowning person falling into a pit.*

(15) 感觉好**压抑**，好像有一团**厚厚**的湿云把你层层包裹，好压抑，好压抑，好想逃，好吵啊!
*It feels so oppressive, as if a **thick** wet cloud is wrapping you layer by layer, so oppressive, so oppressive, I want to escape, it’s so noisy!*

(16) 像我常年活在**冰冷**坭泽中的人，见到阳光也会畏缩的回到我原来的地方。
*Like me who has lived in the **cold** swamp for many years, when I see the sun I will also shrink back to where I was originally.*

(17) 开药吃了后还不好，那我只能认命了，就坠入**地狱**吧!
*If I am still not good after taking the medicine, then I can only accept my fate, and fall into **hell**!*

Several researchers argue that the concept of up-down spatial representation in Chinese has the notion of efficacy: “up” is excellent, moral, and sacred, while “down” is negative, evil, and shameful (Wang et al., 2016). Likewise, McMullen and Conway (2002, p. 181) observe that in “representing our experience of what we call depression in images of descent, we might be compounding the sad affect that appears to be the core of depression with associations of failure and loss of control.”

The Chinese corpus has the main metaphors identified in other contexts (Charteris-Black, 2012; Coll-Florit et al., 2021). Other main CM related to disorder are (18) LIVING ORGANISM (e.g., 怪物*monster*, 催命鬼*death ghost*, 黑狗*black dog*), (19) THING (e.g., 考验*test*, 绳索*ropes*, 脚镣*shackles*), and (20) FORCE (e.g., 反复*repetition*, 重建 *rebuild*). People with depression are conceptualized as energetically deprived in the first two types of metaphors, which imply that their agency has been limited, thus associating disempowerment with a loss of control over their lives; in the latter case, the disorder possesses insurmountable control power, which may be related to the high relapse rate of psychiatric disorders and antidepressant side effects (Cunningham et al., 2021; De Choudhury et al., 2021).

(18) 抑郁症就像一个**催命鬼**跟在你身后甩都甩不掉。我又来了心情很低落无意义感又上来了**黑狗**又来了吗?我真的很累，很累。
*Depression is like a **death ghost** following you and you cannot shake it off. I’m back again, feeling very low and meaningless. Has the* ***black*** ***dog** come again? I’m really tired, very tired.*

(19) 这**绳索**将我牢牢捆绑，没来由的处于要崩溃但不是完全崩溃的阶段
*This **rope** binds me tightly, inexplicably in a stage of collapse but not completely collapsed.*

(20) 不上不下的情绪就卡在这里，坏情绪瓦解的同时又在**重建**，就像是冰山消融的同时却不断凝结；陷入了**死循环**，找不到尽头纾解。
*The mood is stuck here, neither up nor down. The bad mood is disintegrating while **rebuilding**, like an iceberg melting while constantly condensing; trapped in a **vicious circle**, unable to find relief in the end.*

As shown in Table 1, LIFE WITH DEPRESSION is mostly conceptualized through three main source domains: JOURNEY (8.90%), WAR (6.75%), and THEATRE (4.91%).

Our corpus analysis shows that SERIOUS ILLNESS IS WAR is prevalent. The use of WAR metaphors to conceptualize conflict situations and JOURNEY metaphors to conceptualize processes are widely documented in CMT. Metaphors that compare serious illness experiences to WAR are prevalent in support forums, chemotherapy drug websites, and intervention websites. Despite this, previous research has not supported battle metaphors in the context of cancer. Researchers suggest that WAR metaphors ignore social and emotional aspects of healing (Spina et al., 2018), emphasize the importance of treatment at all costs, and present failure to recover as personal failure (Semino et al., 2015). In our corpus, when mapped to the target domain, PEOPLE WITH DEPRESSION are FIGHTERS or STRUGGLERS. DISORDER and INTERPERSONAL RELATIONSHIP are the main objects of the battles (see Table 2). Some depression sufferers feel like they are fighting an invisible and recurrent enemy and are losing the battle due to its high recurrence rate. Interestingly, ME like 挺*persist*, 扛住*survive* seem to help the users retain agency and empowerment when the battle is related to interpersonal relationships (e.g., parents) or sociocultural factors (e.g., prejudice, stigma). Results support Semino et al. (2015)’s assertion that people use BATTLE metaphors in both empowering and disempowering ways, depending on their experiences and preferences.

**Table 2:** Cross-domain mappings of the JOURNEY and WAR metaphors.

Type	Cross-domain mappings	Example
JOURNEY	journey, road, track = > life with depression direction, goal, end, signpost = > life purpose go forward, persevere, cheer = > life roadblock, potholes, trap = > life difficulties	*Life is a lonely journey. Whether you are married or not, you have to achieve self-growth alone and with difficulty.* 人生是一个孤独的旅程。无论结婚与否，你都得独自艰难地实现自我成长。 *The shadows left by the disappointments, disappointments, and failures in the decades of your life miles before you met him* 你在遇见他之前的几十年人生里程中的失望、失意、失败留下的阴影 *I cannot do anything right, I have insomnia every day, I’m irritable every day, the darkness is never ending, and I cannot seem to walk out of it forever* 我什么都做不好，每天失眠，每天烦躁，黑暗永无止境，我好像永远都走不出来了
WAR	Black dogs, demons = > disorder Enemies, stigma, prejudice = > interpersonal relationship Fight, struggle, resist, combat = > treatment Persistence, struggle, survive = > the mindset of fighting mental disorders	*I do not know if I got rid of it. Just suddenly paralyzed with exhaustion, as if I had finished a **battle** that I did not want to**fight** at all.* 不知道自己是不是摆脱了。只是突然累瘫了，好像打完了一场我完全不想打的仗 *Every time I try to get better and study hard there are always inexplicable emotions that appear to **knock me down**.* 每次努力变好，努力学习时总会有莫名其妙的情绪出现把我打倒。 *Every time they start with “you are just like this” and ends with “forget it,” it’s really funny…but I still do not want to give up**fighting**.* 他们每次都以 “你就这样” 开头,“算了吧”结尾，可真逗……但我还是不想放弃战斗啊。

Semino (2017) also point out that the JOURNEY metaphor helps people recognize depression as a process, rather than a fixed condition, and reminds them that they are not alone. As part of our corpus, the JOURNEY metaphors explain how users perceive treatment processes, mental changes, and life challenges (see Table 2). OHC users generally perceive themselves as going through a long, difficult journey with no end. Our data thus confirm that WAR and JOURNEY metaphors shape the subjective experiences of both mental and physical illnesses.

The study finds many THEATRE metaphors, i.e., LIFE WITH DEPRESSION is metaphorically described as 梦境*dream*, 电影*movie*, 舞台*stage*, and 戏剧*drama*, which are absent in other contexts (Charteris-Black, 2012; Coll-Florit et al., 2021). We argue that THEATRE metaphors belongs to the conceptual metaphor EXISTENCE IS VISIBILITY. First, due to the hindrance in cognitive level, interpersonal communication, and motor behavior, patients with depression have a lot of negative self-assessment and rigid self-concern when they have difficulty in real life. They can feel watched and judged, but they also desire to be understood and seen by others (De Choudhury et al., 2013; Refaie, 2014; Yoon et al., 2019). Secondly, people may fear being judged or ostracized for discussing depression in Chinese culture because of the emphasis on collectivism and face. This is because Chinese culture traditionally emphasizes the importance of maintaining one’s public image and avoiding anything that would be seen as a sign of weakness that would damage the collective reputation of the family or community. Studies have supported the concept of sociocentric personhood in relation to mental disorders, as Chinese culture constructs and experiences the disorders in somatic or interperson settings (Xu and Zhang, 2016; Yoon et al., 2019; Zou et al., 2020; Shi and Khoo, 2023). Many people fear appearing burdensome to their families or losing their face if they admit to having depression. As a result, users conceptualize their lives as a disguise. Lastly, THEATRE metaphors resonate with the observation that dream metaphors are prevalent in Chinese stories and legends (Sun, 2022). THEATRE metaphors remind us to pay further attention to the sociocultural connotations of the metaphors.

There are 102 metaphors to describe people with depression, in five categories: CONTAINER (5.21%), MACHINE (4.29%), THING (3.83%), SPLIT SELVES (1.23%) and THEATRICAL ROLE (1.07%).

In SPLIT SELF metaphors, a person suffering from depression is viewed as a divided entity, or as having two different personalities coexisting within them. A healthy self is conceptualized as a long entity that makes us who we are, and a sick self is conceptualized as an ill self (Lakoff, 1993). According to Coll-Florit and Climent (2019), SPLIT SELF metaphors serve as a self-conceptualization tool not only for people with psychotic disorders (such as schizophrenia), but also for those with affective disorders (such as depression). Schizophrenia is characterized by SPLIT SELF metaphors - in keeping with schizophrenia’s literal meaning, split mind.

(21) 抑郁症我的心里好像住着两个**灵魂**一个让我找他人求助，一个让我不要祸害他人一个让我多和朋友沟通，一个让我远离他们一个让我多交朋友，一个让我不要惹是生非一个逼着我我活下去，一个让我闭眼休息.
*Depression. It’s like two **souls** live in my heart. One tells me to seek help from others, one tells me not to harm others. One tells me to communicate more with friends, one tells me to stay away from them. One tells me to make more friends, one tells me not to cause trouble. One forces me to live on, one lets me close my eyes and rest.*

People with depression also present themselves to the world through THEATRICAL ROLE metaphors, such as (22) 小丑 *clown*, (23) 演员 *actor*, and (24) 面具*mask*. They can take on different roles according to their settings, often in an attempt to disguise their true feelings. People with depression turn to fictional characters or theatrical roles that embody their double-sided personalities. The actors within (23) come from a Japanese cartoon novel (*Gin Tama*), in which *Gintoki* is a brave soldier who strives to maintain a peaceful and well-balanced virtual world (*Tendoshu*) against the evil *Oboro* (Darkness). *Matsuyama* is the real persona of an immortal figure called Utsuro, a figure obsessed with death and meaninglessness. To reclaim the meaning of life, *Matsuyama* insisted *Gintoki* decapitate *Utsuro*. People suffering from depression use these examples to express their desire to find inner peace, where the good side must overcome the evil side to remain true and regain meaning in life. Masking is another coping mechanism that involves pretending to be happy and healthy when feeling depressed, by putting on a fake smile, avoiding talking about feelings, or withdrawing from social activities. Masking can make it more difficult to get the help needed. To some extent, THEATRICAL ROLES resonate SPLIT SELF metaphors, where a theatrical role always represents the healthy one, and the *other part* of the self, represented by another theatrical role, is the ill one. It seems that the healthy part of the self is constantly fighting the unhealthy part, which can lead to guilt, self-doubt, self-loathing, and exhaustion.

(22) 我真的思考了很久，发现自己是个**小丑**，外面人模人样，其实里面就是烂人，控制不住自己的废物。
*I really thought about it for a long time and found out that I am a **clown**. I look like a person on the outside, but in fact, I am a rotten person inside, a waste that cannot control myself.*

(23) 我们更要像**银时**一样永不放弃的战斗，打败自己内心深处的**胧**，夺回**松阳**，打败**天导众**在内的阴暗面，拿回我们曾经失去的快乐。
*We must fight like **Gintoki**, never give up, defeat the **Oboro** deep in our hearts, take back **Matsuyama**, defeat the dark side including the **Tendoshu**, and regain the happiness we once lost.*

(24) 大家都在嘲笑我，只有戴着**面具**或者这条路了
*Everyone is laughing at me, only wearing a **mask** or this road is left.*

Repeatedly, depression sufferers conceptualize themselves as MACHINES with weak properties, such as (25–26) 坏 *broken*, 失灵*malfunctioning*, or 低电量 *low battery*, which explain the hopelessness and helplessness they experience (Pritzker, 2003; Refaie, 2014; Yoon et al., 2019). Users also associate themselves with inanimate (27–28) THINGS (e.g., 绿植 *plants*, 废物 *waste*) to condemn their subjectivity and agency. However, MACHINE and THING metaphors tend to emphasize the physical symptoms of depression and ignore its emotional and psychological components. It can reinforce shame and isolation when individuals are conceptualized as defective, broken, or useless. Meanwhile, this finding suggests that metaphors with negative connotations should also be considered as critical self-disclosure attributes, despite the fact that most ready-made analytical tools (such as LIWC) rely on negative adjectives to detect online depressed users (Shi and Khoo, 2023).

(25) 最近药又加了回来，感觉自己变成了只知道开心的单一情绪**机器**
*Recently, the medicine was added back, and I feel like I have become a single-emotion **machine** that only knows happiness.*

(26) 如果我也能像**手机**一样有个充电线就好了，低电量的我什么也做不了，只能维持基本运行。还不知道能坚持多久。
*If only I could have a charging cable like a **mobile phone**, I cannot do anything when my battery is low, and I can only maintain basic operations. I do not know how long I can hold on.*

(27) 我可能像许久没有打理的**房间,**也或许像许久没浇水的**绿植**, 像是抽完的**烟蒂**
*I am like a **room** that has not been taken care of for a long time or maybe like a **green plant** that has not been watered for a long time, like a **cigarette butt** after smoking.*

(28) 我就是一个众所周知的**废物**啊
*I am just a well-known **waste**.*

Conversely, there are fewer metaphors for treatment (4.29%) than for life, people, and disorders. It may be a result of some factors, including the low rate of detection and recovery from depression, the negative outlook on life that many people with depression have, and the lack of treatment options (Kronmüller et al., 2011; Lu et al., 2021; Yao et al., 2022).

### 4.2. INTERPERSONAL RELATIONSHIP metaphors

The main source domains for INTERPERSONAL RELATIONSHIP are (29) REPRESSIVE POWER (4.91%), (30–31) ENEMY (3.68%), (32–33) CONTAINER (2.15%) and (34) SUPPORT (1.53%) (see Table 1).

It is worth noting that FAMILY metaphors are generally negative, and the main source domains include (30) ENEMY, (31) NIGHTMARE, (32) NEGATIVE SOURCE, and (33) SHADOWS, which are extended metaphors for DEPRESSION IS CONTAINER and DEPRESSION IS THEATRE. In DEPRESSION IS CONTAINER, depressed users are like a container of bad emotions, whereas family is the pouring source of the negative emotions. For example, (33) the *shadow* is a part of users’ unconscious mind that contains repressed thoughts, feelings, and desires. In DEPRESSION IS THEATRE, family is the pain maker, the enemy, or the oppressor (which is sarcastically represented as saints in example 30), whereas the depressed sufferers are tragic characters (see Table 3). (30) “Parents are saints” establishes a framework for coordination between parents and saints with their shared qualities of demandingness and supervision. On the contrary, (34) SUPPORT metaphors are applied to OHC and online friends.

**Table 3:** Cross-domain mappings of the FAMILY metaphors.

Type	Cross-domain mappings
CONTAINER	Source of bad mood == > family Pipeline, output == > impact Hell, mire == > family life Containers, burdens == > people with depression
THEATRE	Pain maker, nightmare, clutches == > family Creating painful episodes, scenes == > impact Stage, dream, story background == > family life Clowns, ghosts, dreamer == > people with depression

(29) 真的想逃离这个世界，父母最大的爱好就是否定我，骂我，把我一点点**推进**深渊，然后说一句“废物”
*I really want to escape from this world. My parents’ biggest hobby is to deny me, scold me, **push** me little by little into the abyss, and then call me “**waste**”*

(30) 可恨的这是我的父母。也许我生来就注定活在**炼狱**,而他们则是高高在上的**圣人**，说我是个**小丑**
*It’s hateful that these are my parents. Maybe I was born to live in **hell**, and they are the **saints** above, saying that I am a **clown**.*

(31) 原生家庭是我一辈子逃不出的**噩梦**
*The original family is a **nightmare** that I cannot escape for a lifetime.*

(32) 每次和我妈打完电话都瞬间心如死灰，所有的坏情绪的**源头**我妈能占一半
*Every time I finish a call with my mother, my heart instantly turns to ashes. Half of the **source** of all bad emotions can be attributed to my mother.*

(33) 被嘲笑、被欺凌、被排挤这些经历大概率会变成一辈子都无法摆脱的**阴影**
*Being laughed at, bullied, and excluded are experiences that are likely to become lifelong **shadows** that cannot be escaped.*

(34) 在这个超话里认识了很多新朋友，他们都很温暖，是我的**支柱**，对我很好，希望我们大家都能坚持下
*I have met many new friends in this super talk forum. They are all very warm, they are my **pillars**. They are very good to me. I hope we can all hold on.*

### 4.3. TIME WITH DEPRESSION metaphors

There are 41 TIME metaphors. On the whole, this study confirms the scholars’ conclusions about the time domain representation: Chinese generally presents the metaphorical characteristics of “past in the back and future in the front,” such as 前世*past life*,向前看*looking forward* (Li, 2018; Wang, 2021). Chinese TIME metaphors, such as (35) *behind* and (36) *float back*, are consistent with those in English texts, with an underlying CM of TIME IS MOTION (e.g., the due date has *passed*; there are 3 h *remaining*). In addition, Chinese is more driven by multidimensional time perception, and there is a cultural preference for the representation of time, including more ontological metaphors about time, such as comparing time to (36) 水流 *running water*, (37) 芳华*prime time,* and (38) 花期*flowering season*.Using ontological metaphors allows us to deal rationally with our experiences by recognizing that all concepts are transposable and give abstract concepts concrete form (Lakoff and Johnson, 1980). By identifying time as a separate entity, such as the *flowing season* or the *prime time*, depressed individuals can make sense of their experiences and navigate the complexity of reality.

In contrast to other categories (e.g., PEOPLE WITH DEPRESSION, LIFE WITH DEPRESSION, or INTERPERSONAL RELATIONSHIP), TIME WITH DEPRESSION appears in more idiosyncratic metaphors, rendering it difficult to categorize and discern source and target domains. It is nevertheless important to analyze idiosyncratic metaphors. Firstly, it shows that people may create metaphors to express the experience of TIME WITH DEPRESSION due to its complexity and multifaceted nature. By creating metaphors, they demonstrate their attention and attempt to comprehend and communicate their predicament in online health communities. Secondly, idiosyncratic metaphors might reveal meanings not previously apparent. The imagery associated with it might be vivid and memorable, encouraging the user to think about their issue along these lines. For instance, (37) *prime time* and (38) *flower season* remind users time is brief and fleeting but beautiful and memorable, helping them find meaning and acceptance in difficult times. It can also challenge and motivate them to take action and make positive life changes.

(35) 暴雨，有那么一瞬间希望可以下大点，把我淹了，就不用去想那些**身后**事了，毕竟与我无关……
*Rainstorm, there was a moment when I hoped it could rain harder and drown me so that I do not have to think about the things **behind** me. After all, it has nothing to do with me…*

(36) 如果时光**倒流**的话我一定不要再来到这个世界，哪怕这里有最爱的人。
*If time **floats back**, I will definitely not come to this world again, even if there are people I love the most here.*

(37) 然很想知道自己这辈子的闪光点在哪里，就算是想烟花一样只拥有刹那**芳华**。
*But I really want to know where my shining points are in this life, even if with only **prime time** like fireworks.*

(38) 我日复一日所重复的生活真的有意义吗? 因什么有意义呢? 它的**花期**又会有多久
*Does the life I repeat day after day really make sense? Why does it make sense? How long will its **flowering season** last?*

### 4.4. CYBERCULTURE metaphors

In this paper, CYBERCULTURE metaphors are a distinct type, comprising 20 metaphors. Online health communities (OHC) is a unique genre where jargon and slang are often used. The definitions of many internet buzzwords are lacking in dictionaries or corpora, with no consensus on their meaning. As a result, the source and target domains of CYBERCULTURE metaphors are also difficult to distinguish. CYBERCULTURE metaphors, nevertheless, are characterized by contrasting basic and contextual meanings, such as 树洞*tree holes*, 网抑云 *net suppression cloud*, and躺平 *lay flat*. For example, (40) 网抑云*Net suppression cloud* refers to the widespread posting of depressive content on an app (Neteast Cloud Music) to gain sympathy or attention. The posts can be harmful, as they can normalize depression and make it seem desirable. They can also lead people struggling with depression to feel isolated and alone.

Moreover, CYBERCULTURE metaphors can be used by members of OHC to create a sense of community. For example, (39) 树洞 *Tree hole*, an internet buzzword, refers to a platform on the internet for bearing secrets, and private matters. The term comes from the fairy tale *The Emperor Has Donkey Ears*, which refers to a place where secrets can be told to it without fear of them leaking out.

(39) 每个人都需要一个树洞，存放那些不可轻易示人的秘密、引而不发的情绪、难以启齿的柔弱
*Everyone needs a **tree hole** to store secrets that cannot be easily revealed, emotions that are not easily expressed, and weaknesses that are difficult to speak.*

(40) **网抑云**对于真正抑郁的人来说就是刀子。
***Net suppression cloud** is like a knife to people who are truly depressed.*

## 5. Discussion

Depression may cause persistent sadness, physical pain, shame, negative emotions such as anger and self-loathing, a loss of interest in daily activities, and suicidal thoughts (Yoon et al., 2019). Researchers have found that using first-person singular pronouns, negative emotion words, and death words helps reveal depression symptoms (Liu et al., 2018; Pan et al., 2020). In particular, first-person singular forms can help detect depression since self-references are more frequent among depressed people: a person experiencing physical or emotional pain tends to focus more on themselves and thus use more first-person singular forms (Shi and Khoo, 2023). This paper finds a significant presence of metaphors in OHC that specifically conceptualize the individual with depression. Self-conceptualization metaphors are also a distinct feature of the depressed group: Personal life metaphors accounted for the largest proportion (77.15%), including DISORDER (35.28%), LIFE WITH DEPRESSION (21.93%), and PEOPLE WITH DEPRESSION (15.64%). DISORDER IS DESCENT or CONTAINMENT, LIFE IS JOURNEY, WAR or THEATRE, as well as SELF IS A CONTAINER, MACHINE or THEATRICAL ROLE express feelings of helplessness, loneliness, melancholy, and low agency. There are the fewest metaphors associated with TREATMENT OPTION (4.29%), which in some ways corroborates the observation that groups with depression tend to think negatively (De Choudhury et al., 2021). Alternatively, it may be a result of low treatment rates, slow healing rates, and high relapse rates (Kronmüller et al., 2011; Lu et al., 2021; Yao et al., 2022). Accordingly, negative metaphors should also be considered critical self-disclosure attributes of this population, despite the fact that most ready-made analytical tools (such as LIWC) detect online depressed users using negative adjectives (Shi and Khoo, 2023).

Moreover, we find that container has a different connotation in the Chinese corpus. CONTAINER metaphors can be directly related to SADNESS IS CAPTIVITY (McMullen and Conway, 2002). Other languages also use CONTAINER metaphors heavily. Charteris-Black (2012) argues that CONTAINER metaphors have two connotations: people with depression are conceptualized as containers of negative emotions and confined in enclosed spaces from which they cannot escape. In contrast to LIFE IS CONTAINER (7, 1.07%), the Chinese corpus frequently uses SELF IS CONTAINER (34, 5.21%). The difference probably lies in the value placed on social harmony and face in Chinese culture: people may feel compelled to suppress their emotions, even when feeling negative. Similarly, the Chinese word for depression is *抑郁症*, which translates as *suppressed illness, associating depression* with suppressing one’s emotions. In contrast, the English word *depression* derives from the Latin word *depressus*, which means *pressed down*, meaning people feel weighed down by negative emotions. In a similar vein, the Chinese corpus associates suppressing negative feelings with physical limitations, such as 憋*holding*, 压抑*suffocating*, 奔溃 *collapsing*, and 窒息*breathlessness*.

Our data also indicate two connotations with SELF IS CONTAINER: (1) contextual factors conceptualized as a container from which they feel excluded, primarily due to stigma and discrimination; and (2) interpersonal relationships, particularly family, as a container in which they feel trapped. Contrary to social restrictions and interpersonal difficulties experienced by the OHC users, relational connections are vital for mental health. Individuals and groups can exchange feelings, thoughts, and behaviors through relational connections. People might share inherent resources, including social support, through these connections (Cobb et al., 2010; Myneni et al., 2016; Lei et al., 2022).

Regarding INTERPERSONAL RELATIONSHIP, SUPPORT metaphors are used for (1) the medical treatment and options; and (2) the online health community. According to Coll-Florit et al. (2021) in a Catalan study, most medical diagnosis metaphors are critical of psychiatry and the medical system for limiting the agency and capacity of patients. Medicine is seen as a repressive power that denies individuals with depression agency, the doctor as a prosecutor, or the doctor as a captor who locks them into a diagnosis. Contrary to that, in our corpus, medical treatment is depicted as a source of support and warmth for providing depression sufferers with the necessary assistance and support; or the doctor is seen as a living organism (e.g., 救星 *savior*) for offering hope and vitality. Possibly, this difference can be explained by different medical systems for diagnosis and treatment. Coll-Florit et al. (2021) argue that the Catalan medical system is more problem-focused than person-focused, emphasizing medication and surgery. Antidepressants have many side effects and patients usually have cyclic relapses; therefore patients might lose faith in the medical system. The Chinese medical system, on the contrary, emphasizes balance and harmony through traditional Chinese medicine, which emphasizes mental health as a holistic issue that involves a person’s physical, emotional, and spiritual well-being (Yu, 1995; Pritzker, 2003; Wen and Hua, 2018). Further investigation is needed to understand this difference.

Another group of SUPPORT metaphors is applied to OHC, which demonstrates the value of these communities in helping those suffering from depression, especially when these conditions are stigmatized. In our corpus, many “friends” do not refer to traditional face-to-face friends, but are synonyms for other users of online communities. OHC have become increasingly attractive for social connection due to social deficiencies in offline social circles (Lei et al., 2022). For example, OHC are commonly used by individuals whose primary social network (i.e., friends and family) have limited knowledge of their health condition (Wright, 2016; Liu et al., 2018; Pechmann et al., 2020). The use of OHC can assist users with depression in expressing their emotions and relieving stress. By creating a sense of social belonging, they also assist people with depression to overcome stigma, stigmatization, and social fears (Pan et al., 2020; Lu et al., 2021; Yao et al., 2022).

Users run counter to the negative conceptualization of the family as a source of pain and negative emotions (see Table 3). There are only two instances of FAMILY IS SUPPORT in our corpus, which differs significantly from the prevalence of it in other contexts (Charteris-Black, 2012; Coll-Florit et al., 2021). Possible explanations are: Firstly, Chinese society has a general social expectation of family members’ responsibilities. When people with depression cannot carry out normal social interactions, academic life, and economic output, they may perceive themselves or are seen as “burdensome” to their families. As a result of the wide gap between family expectations and unfulfilled expectations, a feeling of abandonment and deprivation can also occur (Yao et al., 2022); Secondly, depression is associated with low social support, especially when it has long-term adverse effects on close relationships (Kronmüller et al., 2011; Quinlan-Davidson et al., 2021). In particular, Chinese parents of depressed children have poor parenting practices, characterized by low emotional warmth, high denial, harsh punishments, and overprotection (Zou et al., 2020). Family metaphors differ in this way, which reminds us to consider the sociocultural characteristics of the metaphor and implement family-based intervention programs for depressed groups.

In addition, our research finds many THEATRE metaphors, i.e., life is metaphorically described as *dream*, *movie*, *stage* or *drama*, which are absent from other contexts (Charteris-Black, 2012; Coll-Florit et al., 2021). Users frequently use LIFE WITH DEPRESSION IS THEATRE (4.9%), and they describe themselves as actors on stage, performing a part and delivering carefully crafted and rehearsed lines, similar to playing a role in a play. Two cultural connotations are intertwined with THEATRE metaphors. In Chinese culture, there is a strong emphasis on collectivism and face, which may make people feel ostracized or judged if they discuss depression. In non-western cultures, Marsella (1985) notes that depressive disorders and experiences are often expressed without the existential problems associated with Western cultures because non-western tend to construct and experience the disorder in somatic or interpersonal settings. The concept of sociocentric personhood and its relation to mental disorders is also supported by other studies (Xu and Zhang, 2016; Yoon et al., 2019; Zou et al., 2020; Shi and Khoo, 2023). Since Chinese culture emphasizes maintaining one’s public image and avoiding anything that could be perceived as a sign of weakness, it is important to avoid anything damaging the reputation of the family or community. If they admit to depression, many fear appearing burdensome to their families or losing their face. Accordingly, users construct false realities by performing social identities, which are deceptive or hypocritical, as MASK metaphors also imply. Secondly, the THEATRE metaphors describe the concept of EXISTENCE AS VISIBILITY. In the realm of literature study, Clausson (2017) finds THEATRE metaphors are overwhelmingly used in Dashiell Hammett’s *The Maltese Falcon*, suggesting the novel portrays a world where one is trying to see past duplicity, dissimulation, and roleplaying of others while trying to hide one’s own. Due to impairments in cognitive ability, interpersonal communication, and motor behavior, patients with depression have negative self-assessment and rigid self-concern. In addition to feeling watched and judged, they may also worry that others will penetrate their masks to discover what lies beneath and hide signs that might reveal the hidden reality (De Choudhury et al., 2013; Refaie, 2014; Yoon et al., 2019). As a result, they could feel overwhelmed by negative emotions due to the disguise, leading to suppression and repression, which worsen the depression.

Finally, embodied metaphors also reflect the sociocultural characteristics of metaphors. Descriptions of emotional disorders vary according to cultural and historical context. A depressive state may be characterized by somatic symptoms rather than sadness or guilt in some cultures, for example, complaints of nerves and headaches (in Latino and Mediterranean cultures), fatigue, weakness, or imbalance, as well as problems of the “heart” (in Chinese and Asian cultures) or being “heartbroken” (among the Hopi) may all be expressions of depressive states (Marsella, 2002). According to Reali et al. (2016), Spanish culture frames mental illness more as a brain disease than a consequence of psychosocial factors, which influences participants’ initial interpretations of social causal factors and is proposed as a strategy for combating stigma. Two reasons might explain the high proportion of embodied metaphors in the Chinese corpus (30.82%): Firstly, the majority of symptoms of depression are related to physical and mental limitation and pain (Tong, 2020; Taylor-Jackson and Moustafa, 2021). When symptoms are difficult to understand or explain, users process abstract concepts through more familiar simulations of perceptual-motor experience (Poppi and Kravanja, 2019; Beck, 2020). For example, abysses, valleys, and wells are insurmountable, narrow, and dark. The graphic structures of these concrete concepts map the pathological features of depression, including low mood, persistent inhibition of volitional activity, impaired cognitive functioning, learning difficulties, and social impairment (Kauschke et al., 2018); Secondly, embodying metaphors for physical pain also fits with the concept of the mind–body connection in Chinese traditional medicine, which holds that mind and body are interconnected (Pritzker, 2003, 2007). Therefore, any imbalance in either can affect the other. Another proof is that the Chinese corpus associates suppressing negative feelings with physical limitations, such as 憋*holding*, 压抑*suffocating*, 奔溃 *collapsing*, and 窒息*breathlessness*. It remains true, however, that depression metaphors in Chinese and other languages share some sociocultural similarities, such as HAPPINESS IS UP/UNHAPPINESS IS DOWN, JOURNEY, and CONTAINER metaphors, providing a theoretical basis for researching, identifying, and treating psychological disorders in multilingual settings (Charteris-Black, 2012; Coll-Florit et al., 2021).

## 6. Implications and limitations

This section will conclude with methodological, theoretical, and practical implications and suggestions for future research. First, our study has implications for metaphor study, particularly regarding corpus identification of Chinese metaphors and for using the online health community (OHC) as a genre to examine metaphors. Second, a better understanding of how OHC users with depression communicate could boost the effectiveness of counseling and interventions for this population by shedding light on the way they frame and interpret their experiences.

### 6.1. Implication for metaphor study

In this paper, we propose an effective, reliable, and reproducible procedure for metaphor identification in the Chinese corpus (see Figure 1): (1) combine MIPVU to identify ME bottom-up and formulate preliminary working hypotheses; (2) collect more ME top-down in the corpus by performing semantic domain analysis on identified ME; (3) use a reference list to analyze and categorize ME. Its working principle is to identify CM not only on a word-level, but also on the discourse level, which has largely been ignored. From a cognitive perspective, the latter is a key part of the intentional use of metaphors in communication.

In addition, this procedure uses comparison and categorization as paths to explore various target-source pairs: the bottom-up analysis helps to find metaphors based on the comparable similarities between the target and the source, whereas the top-down semantic domain analysis helps trace metaphors based on their categorization path. Both comparison and categorization are possible metaphor processing paths, and the choice depends on the metaphor’s conventionality and linguistic realization (Tong et al., 2021). A metaphor is typically processed through comparison until it becomes conventionalized and processed through categorization, which requires less cognitive effort than comparison. Figure 1 provides an example: ME of 战场*battlefield*. Based on its comparison of basic and context meanings, the bottom-up MIPVU analysis detects it as ME; the top-down analysis expands this category to include geography, location, and theatre. It helps identify more extended metaphors and explores various target-source pairs related to the metaphor.

Culture profoundly affects how people perceive and express their experiences, thoughts, and feelings (Lakoff, 1993; Gibbs and Franks, 2002; Kimmel, 2010; Semino, 2017; Munday et al., 2021). During metaphor processing, emergent meaning is created by interacting with the social and cultural context in which it is used. This research highlights a culturally contextualized approach to metaphorical language in interpreting its connotations. For instance, in cybercultural metaphors, 树洞*tree hole* represent concrete objects without metaphorical meaning in most situations. Another example would be the use of THEATRE metaphors: users describe themselves as actors and perform social identities to construct false, deceptive, or hypocritical realities, which means that the disorder is constructed and experienced within somatic or interpersonal settings where users feel rejected, judged, suppressed, and ostracized. As another example, CONTAINER metaphors in the Chinese Corpus have two connotations: Firstly, contextual factors are conceptualized as containers from which individuals feel excluded, primarily due to stigma and discrimination. Secondly, interpersonal relationships are conceptualized as containers in which individuals feel trapped. These metaphors demonstrate that people are concerned not only with restrictions imposed by the disorder but also with limitations imposed by their sociocultural contexts. Research in the future can also strengthen the integration of metaphorical morphology, coding, phonology, semantics, and pragmatic information from multiple perspectives to provide references for metaphor identification, translation, and interpretation.

Lastly, we contribute to depression metaphor study in a new genre, namely online health communities. Researchers have suggested that bloggers benefit from self-disclosure in maintaining and extending their relationships, which improves their mental well-being (Xu and Zhang, 2016; Lei et al., 2022). By posting on social media, people are less likely to perceive self-disclosure as risky, encouraging them to express themselves openly, vent negative feelings, and seek support from others (Cobb et al., 2010; Potts and Semino, 2017). The lack of social connections within offline social circles or the limited availability of like-minded individuals have made OHC especially attractive for socializing (Ríssola et al., 2019). From several perspectives, our research confirms the value of OHC: (1) Following the findings that OHC are frequently used by individuals whose primary social network (i.e., friends and family) is unaware of their health condition (Wright, 2016; Yoon et al., 2019; Pechmann et al., 2020), users attribute SUPPORT metaphors to OHC and online friends; (2) New metaphors for depression, disorder, and interpersonal relationships have been identified that support the notion that OHC enable people with depression to express themselves freely and provides a better basis for analyzing their conceptualizations. Our findings are in line with the proposals of Coll-Florit et al. (2021) and Semino et al. (2015), and a wide range of other researchers that online discourse genres can enable those in need to regain a sense of control over their lives and emotions, and to alleviate their suffering (Potts and Semino, 2017; Lu et al., 2021; Lei et al., 2022). As we will demonstrate shortly, OHC and its metaphorical language also have implications for mental health management.

### 6.2. Metaphors for mental health management

Metaphors can be a very powerful tool in healthcare. As metaphors facilitate access to and symbolize emotions, uncover and challenge tacit assumptions, and introduce new frames of reference, they play a significant role in constructing and expanding alternative perspectives (Tay, 2017). By providing relevant theoretical nuances derived from linguistic studies of metaphors, we find ways to emphasize more meaningfully the inherent cross-disciplinarity of psychotherapeutic metaphor research. Therapeutically relevant topics correspond to target domains, conceptual resources used to describe them correspond to sources, and how the latter is understood to be related to the former corresponds to cross-domain mappings (Tay and Neimeyer, 2021). Joining psychophysical and discourse analytical points of view, we will analyze the implications of metaphor research based on source-target connections at the three levels found in this study: embodied metaphors, sociocultural metaphors, and idiosyncratic metaphors.

First, depression metaphors present a high proportion of embodied metaphors, probably because somaticization symptoms associated with depression are common (Coll-Florit et al., 2021; Cunningham et al., 2021; De Choudhury et al., 2021). The ABYSSES, VALLEYS, and WELLS are difficult, narrow, and dark, which correspond to the pathological features of depression, including depressed mood, persistent inhibition of voluntary activity, impaired cognition, and social impairment. Embodied metaphors are unlikely to misunderstand, and Semino et al. (2016) note that embodied metaphors can facilitate some form of embodied simulation in the addressee, which may lead healthcare professionals to provide empathic responses. Psychiatrists and health service practitioners should therefore consider embodied metaphors as essential languages for practice, develop corresponding standards, especially linguistic specification of metaphorical terms for interventions and treatments, and devise strategies to enhance their ability to analyze metaphors. For example, psychiatrists can send sunny and warm pictures to visitors on cloudy days as part of psychological intervention programs, collect somatization health information with somatic suits, or analyze embodied metaphors in visitors’ online self-disclosure texts. An example relevant here is Tay’s (2014) analysis of metaphors used by earthquake victims to describe their trauma. Earthquake victims used the metaphors “the ground was still moving” and “it was dark” to describe their bodily experiences of the earthquake. These metaphors are examples of target domains because they are used to describe the physical sensations of the earthquake. A chain-like dynamic emerged, however, as the interaction progressed, where topics transformed from targets to sources, which were creatively exploited as source domains for other aspects of victims’ trauma, such as their uncertainty about the future (for example, *we did not know where the future was going*, and *we were in the dark*). The “chaining” dynamic is possible because metaphors are embodied, which means they are grounded in our bodily experiences, making them flexible and creative. In a recent study, Tay (2023) has further argued that topic-triggered metaphors have source domains whose pragmatic motivations are driven by the topic rather than some ostensibly static conceptual metaphorical structure. It has implications for evaluating the use of embodied metaphors in health-related contexts because topic-triggered metaphors enable people to analyze metaphors in terms of their impact on patient outcomes.

Furthermore, we have discussed metaphors on a sociocultural level, where culture-specific knowledge and experience are crucial to constituting, interpreting, and fully appreciating the source domain. The most disabling mental illness, depression, is associated with low social support, especially when it has long-term adverse effects on close relationships (Pan et al., 2020; Pechmann et al., 2020). Identifying depression metaphors in the Chinese corpus pinpoints the sociocultural environment people with depression are experiencing: lack of offline support, social stigmatization, and substitutability of offline support with online support. Practitioners can incorporate metaphor analysis into parenting education, family therapy, and community support groups. Family-based interventions can help practitioners address various issues, including child maltreatment, substance abuse, and mental health concerns. A metaphor with social-cultural connotations is always elaborate, individualistic, mini-anecdotes. It contains a richer dramatic content and contextualization, allowing the therapist and client to construct a scenario with its internal logic, encouraging the client to “think their thinking” and to find new ways of approaching old problems through analogy (Stott et al., 2010). Therapists can, for example, assist the client in explicitly developing a range of scripts from his/her THEATRE METAPHORS and aid him/her in recognizing the arbitrariness of those scripts and their loose relationship to reality. Moreover, there may be a greater opportunity for effective communication between AI robots and depressed users, reducing the stigma associated with mental health issues and encouraging people to seek help. By providing personalized feedback and responses, AI robots can help reduce feelings of isolation and help users cope with depression by providing a safe space for people to interact and share their feelings without judgment. Developers of companion robots, electronic chat rooms, and chatbots should incorporate metaphors into their scripting language to provide “appropriate” answers to more obscure conversations that contain metaphors. Meanwhile, we also provide new evidence supporting the potential benefits of OHC for depression, as friends and contacts in online support communities may indicate a higher level of social integration, leading to greater social support (Pan et al., 2020; Lu et al., 2021). The substitutability of offline illness work may be particularly helpful to those without or with limited access to offline support. Based on our metaphor types, OHC organizers can develop metaphorical images, audio, and emojis to increase users’ willingness to participate, help them cope with loneliness and anxiety, and get emotional support.

Finally, this study finds that some source-target connections are built at the idiosyncratic level, at which individuals’ knowledge and experiences are unique (Tay, 2017). In the Chinese corpus, there is a user who eloquently describes depression, which translates as: “When anxiety and depression come, try first notice the thoughts and ideas in the brain, do not judge, do not entangle, then imagine, put ‘them’ in a drift bottle, seal it, watch this ‘disease-filled’ drift bottle slowly move away along the tide and disappear on the horizon.” (当焦虑和抑郁来袭得时候, 可以试试, 先觉察到脑中的思绪和想法, 不评判, 不纠缠, 然后想像一下, 把“他们”装在一个漂流瓶里, 封住, 注视着这个装了“病”的漂流瓶顺着海水慢慢离自己而去, 消失在天边). Unlike the conventional DEPRESSION IS CONTAINER, the user draws on a novel metaphor that creatively depicts the container as a floating bottle. She uses extended metaphors to describe how she unravels the bad emotions by letting the bottle float away with the tide. This image may resonate with her experience of using a floating bottle to release deep-buried psychological loneliness and depression. In a similar instance, Tay (2015) documents a case in which a therapist and client used metaphors that shared an underlying embodied inferential structure of “moving out of a physical container,” facilitating the development of a collaborative framework to solve a problem. Tay (2017) argues that since therapists cannot contribute substantive details at the idiosyncratic level, allowing clients to play an active role in facilitating their own betterment may increase their sense of empowerment and agency. Additionally, individuals can incorporate metaphors into self-care through writing, painting, singing, or other forms of multimodal discourse, expressing emotions, obtaining support, and building communication channels.

This study still has some shortcomings. Most microblog users are young and middle-aged, so this study’s results may not apply to elderly population with depression. Conducting a follow-up study to examine metaphors used in elder groups and how metaphor research can be applied to aging issues would be beneficial (Landau et al., 2018; Malkomsen et al., 2021). The second limitation of this study is that it analyzes linguistic data alone and does not include multimodal data, such as pictures, videos, and music. Particularly, metaphors are related to visual stimuli, gesture analysis, and multimodal representations such as films and advertisements (Potts and Semino, 2017; Poppi and Kravanja, 2019; Semino, 2021; Pérez-Sobrino et al., 2022). Metaphors influence human conceptualization beyond the canonical verbal and textual cues, and metaphors also affect other representational modalities.

## 7. Conclusion

In this paper, we propose an effective, reliable, and reproducible procedure for metaphor identification in the Chinese corpus: (1) combine MIPVU to identify metaphorical expressions (ME) bottom-up and formulate preliminary working hypotheses; (2) collect more ME top-down in the corpus by performing semantic domain analysis on identified ME; (3) analyze ME and categorize conceptual metaphors using a reference list. In this way, we have gained a greater understanding of how depression sufferers conceptualize their experience metaphorically, in an under-represented language in literature (Chinese) of a new genre (online health community). We confirm a number of depression metaphors in other contexts and compare them with the Chinese metaphors, providing a theoretical basis for researching, identifying, and treating psychological disorders in multilingual settings. We find sociocultural attributes of Chinese metaphors that represent constraint and containment, especially in terms of THEATRE and CONTAINER metaphors, that portray the disorder and the lack of offline interpersonal support. As a result, this study confirms the potential of online health communities to serve as a metaphor genre for facilitating users’ expression, alleviating social fear and stigma, and assisting them in seeking social support. Our study also identifies new metaphors with source-target connections based on embodied, sociocultural, and idiosyncratic levels, which were not previously considered in depression metaphor research. We have examined metaphor research’s theoretical and practical implications at these three levels, emphasizing its inherent cross-disciplinary nature in meaningful ways.

## Data availability statement

The original contributions presented in the study are included in the article/supplementary material, further inquiries can be directed to the corresponding author.

## Author contributions

JS: conceptualization, methodology, investigation, project administration, writing, review and editing. ZK: methodology, formal analysis, and editing. All authors have read and agreed to the published version of the manuscript.

## Funding

This study was funded by The National Social Science Fund of China (Grant No: 20CYY016).

## Conflict of interest

The authors declare that the research was conducted in the absence of any commercial or financial relationships that could be construed as a potential conflict of interest.

## Publisher’s note

All claims expressed in this article are solely those of the authors and do not necessarily represent those of their affiliated organizations, or those of the publisher, the editors and the reviewers. Any product that may be evaluated in this article, or claim that may be made by its manufacturer, is not guaranteed or endorsed by the publisher.

## Footnotes

^1^see: https://ucrel.lancs.ac.uk/wmatrix/
